# Influence of selected dosages of plastic microparticles on the porcine fecal microbiome

**DOI:** 10.1038/s41598-024-80337-x

**Published:** 2025-01-08

**Authors:** Ismena Gałęcka, Andrzej Rychlik, Jarosław Całka

**Affiliations:** 1 Department of Epizootiology, Faculty of Veterinary Medicine, University of Warmia and Mazury in Olsztyn, Oczapowskiego 13, 10-719 Olsztyn, Poland; 2 Department of Clinical Diagnostics, Faculty of Veterinary Medicine, University of Warmia and Mazury in Olsztyn, Oczapowskiego 14, 10-719 Olsztyn, Poland; 3 Department of Clinical Physiology, Faculty of Veterinary Medicine, University of Warmia and Mazury in Olsztyn, Oczapowskiego 13, 10-719 Olsztyn, Poland

**Keywords:** Metagenomics, Microplastic, Next-generation sequencing (NGS), Pig, Polyethylene terephthalate (PET), 16s rRNA, Public health, Microbiology

## Abstract

Studies conducted so far have shown that nano- and microplastic may disturb the intestinal microenvironment by interacting with the intestinal epithelium and the gut microbiota. Depending on the research model used, the effect on the microbiome is different—an increase or decrease in selected taxa resulting in the development of dysbiosis. Dysbiosis may be associated with intestinal inflammation, development of mental disorders or diabetes. The aim of the study was to analyze the intestinal microbiome in 15 gilts divided into 3 research groups (n = 5; control group, receiving micropartices at a dose 0.1 g/day (LD) and 1 g/day (HD)). Feaces were collected before and after 28 days of exposure to PET microplastics. The analysis of the intestinal microbiome was performed using next-generation sequencing. Alpha and beta diversity indices were compared, showing, that repetition affected only the abundance indices in the control and LD groups, but not in the HD group. The relationships between the number of reads at the phylum, genus and species level and the microplastic dose were calculated using statistical methods (*r*-Pearson correlation, generalized regression model, analysis of variance). The statistical analysis revealed, that populations of *Family XIII AD3011 group*, *Coprococcus*, *V9D2013 group*, *UCG-010* and *Sphaerochaeta* increased with increasing MP-PET dose. The above-mentioned taxa are mainly responsible for the production of short-chain fatty acids (SCFA). It may be assumed, that SCFA are one of the mechanisms involved in the response to oral exposure to MP-PET.

## Introduction

The ubiquitousness of plastic in everyday life makes scientists emphasise that we are living in the plastic age^1^. The formation of plastic microparticles is one of the many adverse aspects of this material’s use. Microplastic (MP—particles of less than 5 mm in size) has been identified in surface waters in the northern and southern hemispheres^2^, in samples collected in high mountain ecosystems^3^, in vegetables and fruit^4^ meat^5^ and even in human blood^6^. These data show, that—due to human activity—microplastic has reached every corner of the globe, and it can be regarded as ubiquitous. Plastics vary in terms of their physicochemical properties. Polyethylene terephthalate (PET) is a polyester used in industry mainly to make food packaging, especially bottles for beverages, electric device casings or textiles. It is used practically in every element of the food production chain^7^. Food packaging may be an important MP source in food chain^8–10^. Next to polypropylene (PP), PET was the most frequently found plastic in drinking water^11^. Winkler et al. have shown that repeated opening and closing of plastic bottles can contribute to releasing microparticles into bottled water^12,13^. The presence of MP in animal (including pig) manure^14^ confirms that organic fertilisers may be a source of MP in soil, from where they can infiltrate vegetables and fruit^4^ or raw materials for feed production^15^. Therefore, this may result in recirculation or even accumulation of microplastic in the food (in products derived from plants and animals). The domestic pig (*Sus scrofa domestica*) is an animal species bred mainly for meat and offal. Feeding animals with feed contaminated with plastic microparticles^9,16^ may contribute to their entering the food chain and, ultimately, being consumed by humans. Pork can also be contaminated at a later production stage, e.g. in packing^17^ or in meat being processed, for example, on plastic boards^18^. Utilization of a domestic pig as a research model involves two aspects: (1) examining the effects on livestock as part of the food chain and (2) on the potential effects on humans as a model in biomedical research. The anatomical and physiological similarity shared between humans and pigs establish the digestive tract of these animals as a valuable model for biomedical research^19^. The observed changes in porcine physiology potentially serve as a research model to explain analogous processes occurring within the human gastrointestinal system.

Currently, there is limited information in the literature on the effect of PET microparticles on the domestic pigs. So far, it has been investigated, that polystyrene can affect pig production by reducing daily gains and impairing meat quality^20^. Analogous changes have been detected in chickens^21^. It was found, that MP can accumulate in the muscles and also cause deterioration of the quality of muscle tissue^21^.

The gut microbiota is a set of bacteria, archaea, fungi, protozoa and viruses^22^. Although the role of microorganisms and genes included in the microbiome has not been explained sufficiently, it is already known that it can play a significant role in the host’s body^23–30^. The alimentary tract is one of the main routes of exposure to microplastic^4,10,13^ and the gut microbiota is particularly exposed to it. The gut microbiome mediates nutrient metabolism, performs protective functions, and is responsible for the host’s health status by modulating its immune response^23,26,29–33^. The microbiome composition is affected by diet, age, pathogens, antibiotic use and the birth mode^32,34–39^. Any modifications to the microbiota composition caused by pathological agents, e.g. infection with *Escherichia coli*, can contribute to a decrease in production results, e.g. by causing diarrhoeas or worse nutrient absorption from feed^33,34,40^. Exposure to microplastics and changes in the microflora may therefore be associated with deterioration of breeding indicators^21^, accumulation in tissues^41^ or deterioration of the animals health status^42^. This might have an impact on the breeding, value and safety of animal products.

Studies conducted so far have shown that nano- and microplastics can disturb the intestinal microenvironment by interacting with the intestinal epithelium and the gut microbiota^41,43–49^. Microplastics contributed to a reduction of alpha diversity in the chicken^48^ and human^50^ microbiome and to changes in some bacteria count and the gut microflora structure^48^. A single exposure to microplastics can have a significant impact on the gut microbiome by inducing the growth of opportunistic bacteria and reducing beneficial taxa^51^. Depending on the experimental design, the results indicated an increase^51^ or decrease^52^ in the population of the Firmicutes and Bacteroidota. Microplastic may also be covered with biofilm, contributing to disturbances in the microbiological balance of a studied ecosystem^53^. There may also be functional MP degradation genes among bacteria, which currently represent hope for effective MP biodegradation^53,54^.

Therefore, it is important to determine the impact of MP on the microbiome in different taxa and to identify taxa susceptible to MP. Since the current exposition of the population to microplastic is indisputable, significant studies of microplastic effects on the microbiome seem to be desired. The current study could provide information important to public health safety. The aim of this research was to determine the impact of two selected doses (0.1 g/day and 1 g/day) of PET microplastic (MP-PET) on porcine faecal microbiome. At present, the scientific literature lacks data regarding the influence of PET microparticles on the fecal microbiome of domestic pigs.

## Results

### Animal body weight

**Fig. 1:**
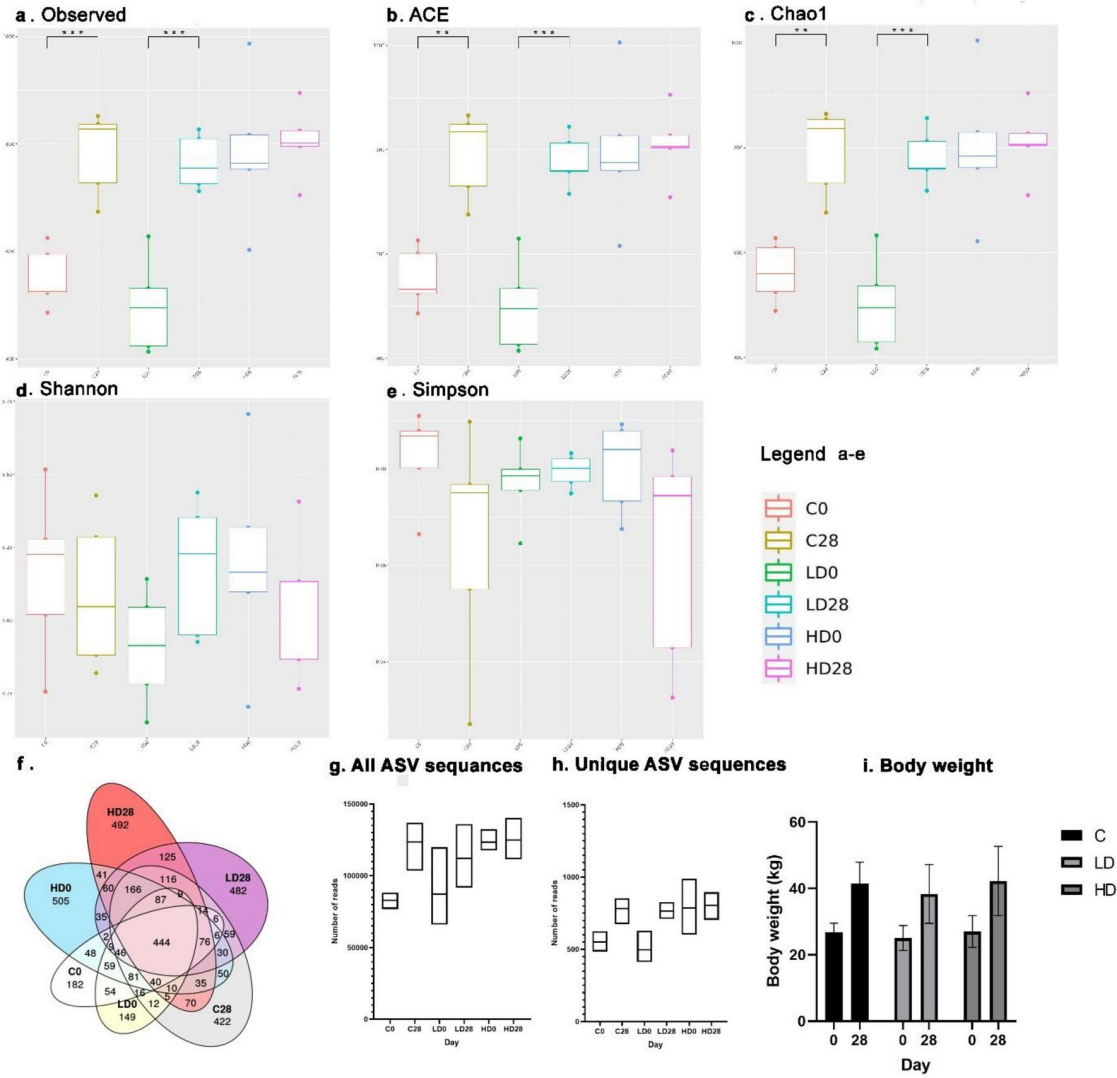
Alpha diversity in all experimental groups and body weight. Within sample diversity measured by (**a**) Observed, (**b**) ACE, (**c**) Chao1 (**d**) Shannon and (**e**) Simpson index. (**f**) Venna diagram shows the number of shared and individual OTUs in all groups. (**g**) Sum of counts of all and (**h**) unique ASV sequences in the study groups. (**i**) Gilts’ weight during the experiment. The gilts initial and final body weights were taken into account. C0—control group day 0, C28—control group day 28, LD0—low dose group day 0, LD28—low dose group day 28, HD0—high dose group day 0, HD28—high dose group day 28). Only statistically significant differences between the repetitions in groups C, LD and HD were included in the graph **p < 0.01, ***p < 0.001.

The body weight measured every week did not show any statistically significant differences between the experimental groups (*p* > 0.05). The data presenting the initial and the final animal body weight are shown in Fig. 1i. No diarrhea or changes in the appearance of faeces, or deviations in the amount of consumed fodder or water were observed throughout the experiment.

### Alpha and beta diversity

Of the identified 3938 OTUs, 444 were present in all the groups, and a total of 2232 were unique OTUs for C0-182, C28-422, LD0-149, LD28-482, HD0-505 and HD28-492. The Venna diagram shows the interdependence of OTU presence in all study groups (Fig. 1f). A total of 3,270,352 total ASV sequences were identified, of which between 498,200 ± 876,054 (LD0) and 804,600 ± 681,711 (HD28) were unique ASV sequences for the group (Fig. 1g,h). The lowest ASV was recorded in the C0 group and the highest in the HD28 group.

An alpha diversity analysis revealed the presence of statistically significant differences (*Mean* ± *SD*) in the Observed index (C0 550.6 ± 57.03 vs. C28 783.80 ± 78.43 and LD0 498.20 ± 87.61 vs. LD28 766.00 ± 50.73), Chao1 (C0 562.04 ± 57.37 vs. C28 792.79 ± 83.69 and LD0 502.20 ± 88.03 vs. LD28 781.32 ± 53.65) and ACE (C0 554.04 ± 57.64 vs. C28 790.85 ± 83.32 and LD0 500.02 ± 87.52 vs. LD28 778.14 ± 50.58) between groups C0 vs. C28 and LD0 vs. LD28. The Shannon and Simpson indices in every comparison did not show statistically significant differences (*p* > 0.05). The results are shown in Fig. 1a–e. No differences in Observed, Chao1 and ACE indices were found between groups HD0 vs. HD28 and between groups C28 vs. LD28 vs HD28. According to them, MP-PET did not have any impact on microbiota diversity but only on its abundance, which was greater in the group C and in those receiving a low dose compared to those receiving a high dose. Statistically significant differences in abundance indices were observed at *p < 0.05 between C0 vs. HD0, **p < 0.01 between LD0 vs. HD28 and ***p < 0.001 between C0 vs. HD28, C0 vs. LD28, C28 vs. LD0 and LD0 vs. HD28. In order to better visualize the potential changes induced by MP-PET exposure, these differences are not included in Fig. 1a–e.

PCA and PCoA analysis based on the Bray–Curtis distance were used to examine the clustering of samples. The PERMANOVA results showed that neither the microplastic dose applied (*p* > 0.05 for C28 vs. LD28 vs. HD28) nor a replicate (*p* > 0.05 for C0 vs. C28, LD0 vs. LD28 and HD0 vs. HD28) had an impact on the beta diversity (Supplementary Fig. S1a–h). Differences at the *p* < 0.05 level are included in Additional Table S1 and apply to other comparisons between groups.

### Microbiome composition at phylum level

**Fig. 2:**
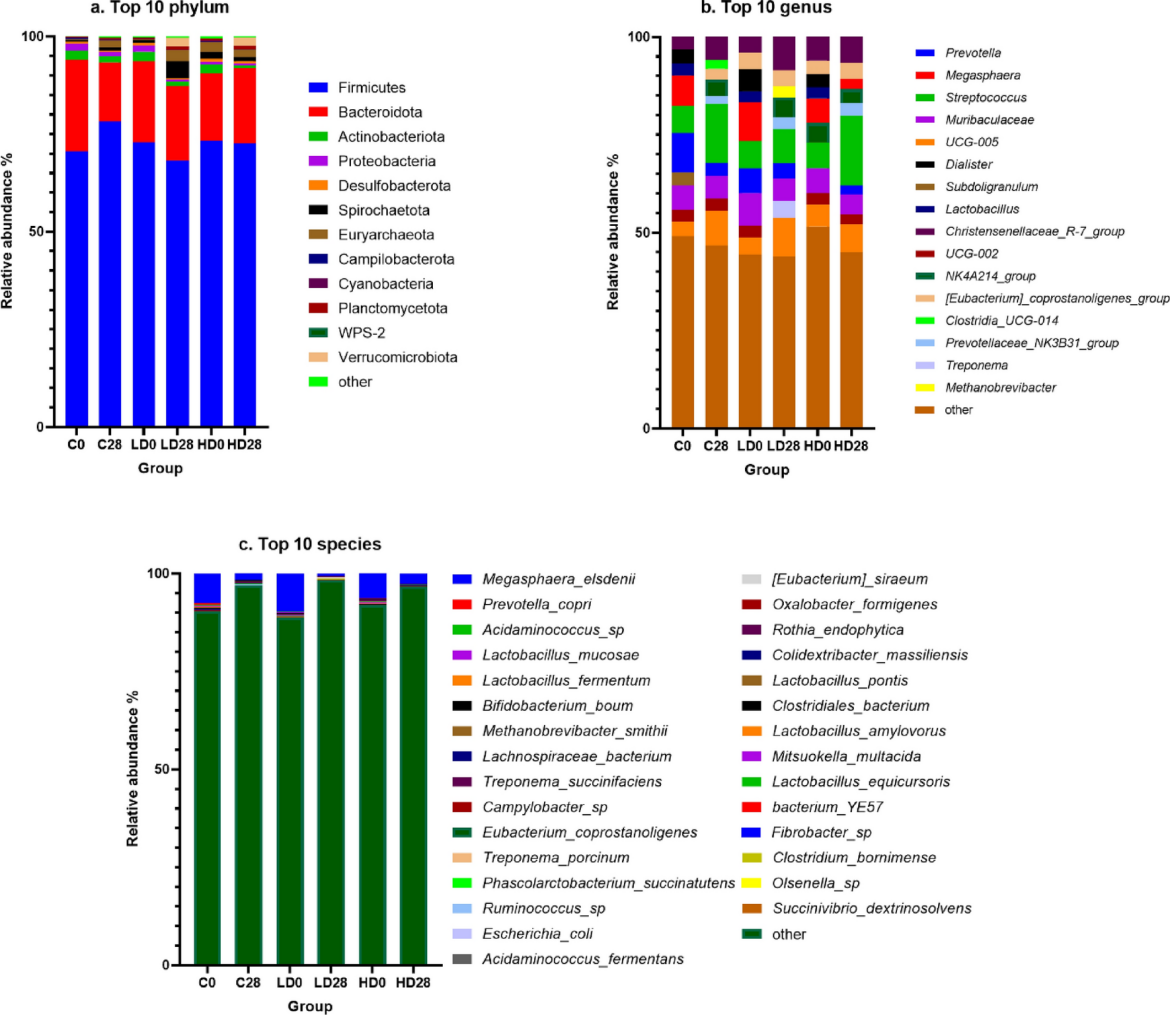
The composition analysis of the microbiota community at the (**a**) phylum, (**b**) genus and (**c**) species level. The graphs show ten of the most numerous (**a**) phyla, (**b**) genera and (**c**) species. C0—control group day 0, C28—control group day 28, LD0—low dose group day 0, LD28—low dose group day 28, HD0—high dose group day 0, HD28—high dose group day 28.

Firmicutes and Bacteroidota accounted for the largest population in all the research groups—over 90% of the total bacteria population except in group LD28 (Firmicutes: C28 (78.22%), HD0 (73.32%), LD0 (72.88%), HD28 (72.65%), C0 (70.59%), LD28 (68.24%) and Bacteroidota: C0 (23.42%), LD0 (20.74%), HD28 (19.21%), LD28 (19.12%), HD0 (17.19%), C28 (15.16%)). Phyla identified with a relative population size exceeding 1% include Actinobacteriota Proteobacteria, Euryarchaeota, Proteobacteria, Spirochaetota, and Verrucomicrobiota (Supplementary Materials Table S2). The ten most frequently found phyla for all the groups were selected and shown in Fig. 2a. The most significant disparity was noted in the Firmicutes count after 28 days in group C, exhibiting an increase of 7.62%. MP-PET contributed to a reduction in the low dose (LD) group by 4.64% and in the high dose (HD) group by 0.66%. Bacteroides decreased in group C by 8.26% and in LD by 1.63%, while the high dose of MP-PET augmented its abundance by 2.01%. Analyzing the composition of the ten most prevalent phyla on days 0 and 28, discernible differences were observed. In group C, the phyla remained the same; however, variations were noted in the relative abundance of identified taxa, particularly evident in Euryarchaeota, with a substantial increase from 0.29 to 1.76%. In the LD group, Euryarchaeota increased from 0.18 to 2.82%. Conversely, the HD group exhibited a reduction of 0.52% after 28 days. The relative abundance of WPS-2 decreased in the LD group, and after 28 days, it was no longer among the ten most frequently identified phyla, being supplanted by Verrucomicrobiota. A similar transition occurred in the HD group, where Verrucomicrobiota replaced Cyanobacteria. In all groups, there was a reduction after 28 days in Actinobacteriota (by 0.86% (C), 1.40% (LD), and 1.64% (HD)), Proteobacteria (by 0.55% (C), 1.18% (LD), and 0.07% (HD)), and Desulfobacterota (by 0.38% (C), 0.15% (LD), and 0.27% (HD)). A change trend similar to Euryarchaeota was observed in the case of Cyanobacteria and Spirochaetota, with an increase in the C and LD groups and a decrease in the HD group.

### Microbiome composition at the genus level

The difference in bacteria compositions depends on the study group. *Prevotella* were the most numerous in group C0 (9.96%), *Streptococcus* in HD28 (17.79%), C28 (15.11%), HD0 (6.50%), *Megasphera* in LD0 (9.95%), and *UCG-005* LD28 (9.95%). Genera identified with the relative population size in excess of 1% include *[Eubacterium] coprostanoligenes group, Akkermansia, Alloprevotella, Catenibacterium, Christensenellaceae R-7 group, Clostridia UCG-014, Clostridium *sensu stricto* 1, Coprococcus, Dialister, Faecalibacterium, Lachnospiraceae AC2044 group, Lachnospiraceae XPB1014 group, Lactobacillus, Methanobrevibacter, Mitsuokella, Muribaculaceae, NK4A214 group, Olsenella, Oscillospira, p-2534-18B5 gut group, Prevotella, Prevotellaceae NK3B31 group, RF39, Rikenellaceae RC9 gut group, Roseburia, Ruminococcus, Shuttleworthia, Subdoligranulum, Succinivibrio, Succinivibrionaceae UCG-001, Treponema, UCG-002 and UCG-010* (Supplementary Materials Table S2). Figure 2b shows the ten most frequently found genera for all the groups. The analysis of the ten most common taxa revealed the absence of *Prevotella* only in the HD0 group. However, further analysis revealed that with an increase in the dose of MP-PET, there was a smaller reduction in the relative abundance of these bacteria (by 6.72% (C), 2.49% (LD), and 0.35% (HD)). In the control group, it was noted that the genera *Christensenellaceae R-7 group*, *Muribaculaceae*, *Prevotella*, *Streptococcus*, *UCG-002*, and *UCG-005* were the ten most common genera on day 0 and day 28. After 28 days, differences were only noted in the percentage of mentioned taxa. In the LD group, similar to group C, the same six genera constituted the core of the microbiome on both days 0 and 28 (*[Eubacterium] coprostanoligenes group*, *Christensenellaceae R-7 group*, *Muribaculaceae*, *Prevotella*, *Streptococcus*, and *UCG-005*). The HD group was more stable, as eight of ten genera were recorded on both days 0 and 28 (*[Eubacterium] coprostanoligenes group*, *Christensenellaceae R-7 group*, *Megasphaera*, *Muribaculaceae*, *NK4A214 group*, *Streptococcus*, *UCG-002*, and *UCG-005*). The largest difference at this level was observed in the case of *Streptococcus* bacteria, where in the HD group, it increased by as much as 11.28%, in the C group by 8.17%, and in the LD group by 1.91%. Interestingly, the relative abundance of *Megasphaera* under the influence of a low dose of MP-PET was significantly reduced. From the dominant taxon (9.95%) on day 0, on day 28, it constituted only 0.59% of the population of all identified genera. The high dose of MP-PET, on the other hand, resulted in a reduction of only 3.72%. In group C, the reduction of *Megasphaera* was 6.02%.

### Microbiome composition at the species level

*Megasphaera elsdenii* was the most frequently identified bacteria species, and it was the only one to achieve a relative population size of more than 1% LD0 (9.75%), C0 (7.60%), HD0 (6.23%), HD28 (2, 57%), C28 (1.73%) except LD28 (0.57%). The percentages reduction in the groups were as follows: C—5.88%, LD—9.18%, and HD—3.65%. The other species accounted for less than 1% of the relative population size. Figure 2c shows the ten most frequently found species for all the groups. Full descriptive statistics of the results (relative population size ≥ 0.01%) are shown in Supplementary Materials Table S2.

### Analysis of variance

**Fig. 3:**
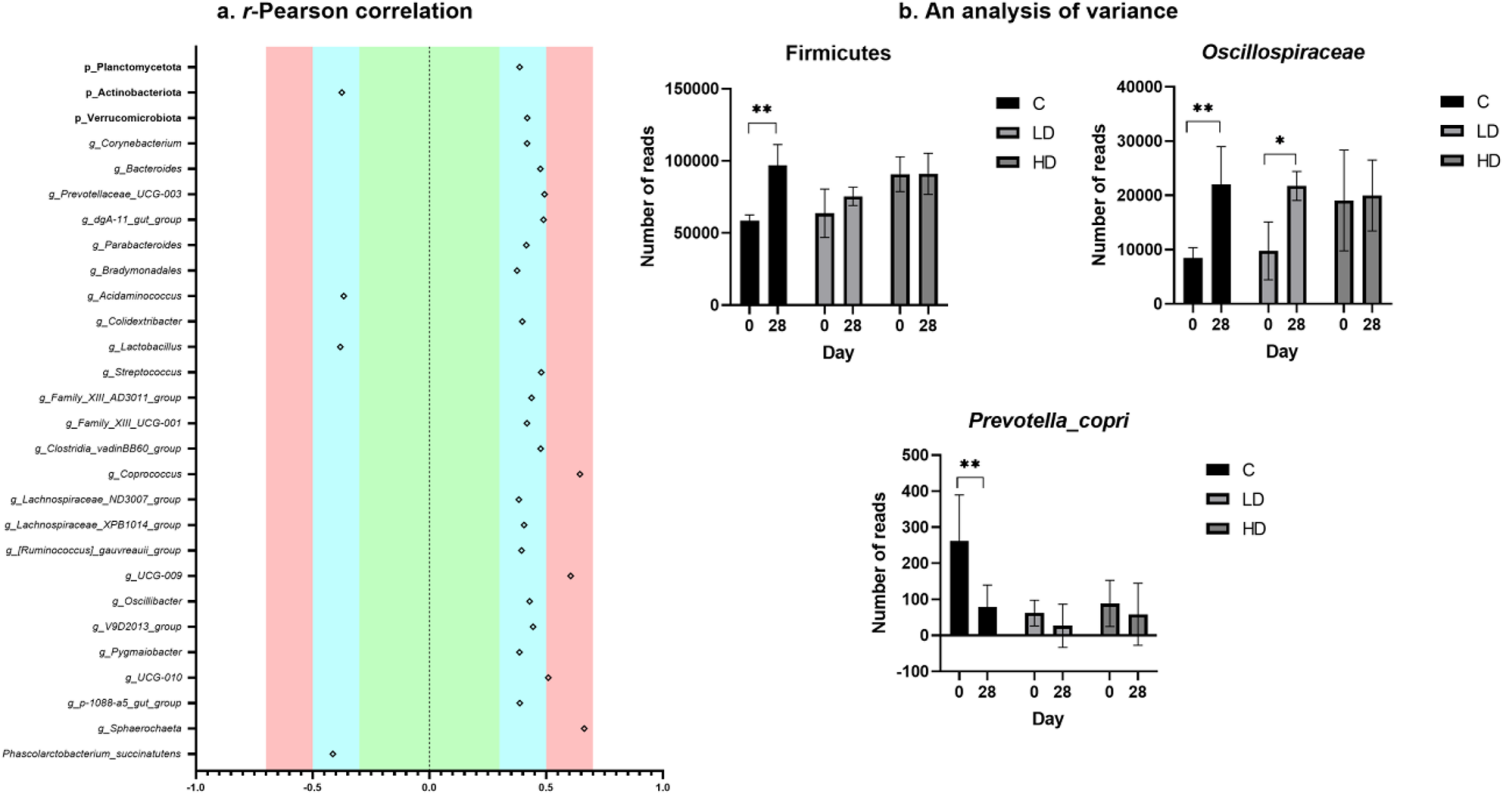
(**a**) *r*-Pearson correlation between the dose of MP-PET and (**b**) an analysis of variance for selected bacteria. (**a**) *r*-Pearson correlation between the dose of MP-PET and the number of readings at the level of phylum, genus and species. The calculated r values were interpreted as follows: 0–0.3 = weak positive/negative correlation (green); 0.3–0.5 = moderate positive/negative correlation (blue); 0.5–0.7 = strong positive/negative correlation (red); 0.7–1 = very strong positive/negative correlation (white). (**b**) An analysis of variance for selected bacteria (*Mean* ± *SD*), where statistically significant differences were demonstrated (*p*<0.05) in the number of readings depending on the MP-PET dose. C – control group, LD – low dose group, HD – high dose group.

The analysis of variance showed that for Firmicutes, *Oscillospiraceae* and *Prevotella copri*, statistically significant differences were demonstrated (*p* < 0.05) in the number of reads, considering the MP-PET dose and the replicate (Fig. 3b). The dose of 1 g/animal/day was not found to change significantly the number of readings for Firmicutes (from 90,744.60 ± 12,019.79 to 91,051.40 ± 14,293.17), whereas the population size was found to increase (from 63,715.00 ± 16,739.84 to 75,399.00 ± 6362.51) at the dose of 0.1 g/animal/day, although not as significantly as in the group C (from 58,521.80 ± 4082.86 to 96,847.00 ± 14,514.74). Similar changes were noted at a high dose of MP-PET in *Oscillospiraceae* (from 19,074.40 ± 9279.81 to 19,974.40 ± 65 34.21) although the same increasing trend was noted in the control group and in that receiving a low dose (from 8435.00 ± 1948.38 and 9782.60 ± 5317.86 to 22,026.60 ± 6953.83 and 21,758.80 ± 2666.42). For *Prevotella copri*, the population size was seen to decrease in all the groups, but the reduction correlated with increasing MP-PET dose (C0 262.20 ± 127.56, LD0 62.00 ± 35.75, HD0 88.60 ± 63.94 and C28 78.60 ± 60.99, LD28 26.80 ± 59.93, HD28 58.80 ± 86.07). Moreover, 109 taxa were demonstrated (phylum = 4, class = 7, order = 14, family = 20, genus = 53 and species = 11), where statistically significant differences were noted between groups 0 and 28.

### *r*-Pearson correlation

Moderately negative (n = 4), moderate (n = 20) and strong (n = 4) correlations were shown between the microplastic dose and the number of bacteria readings at the phylum, genus and species level (Fig. 3a). The other results were regarded as statistically insignificant. For the moderately negative correlation, the r values ranged from *r* = − 0.41 (*Phascolarctobacterium succinatutens*) to *r* = − 0.38 (Actinobacteriota). The *r* values for the moderate correlation from *r* = 0.39 (*Pygmaiobacter*) to *r* = 0.49 (*Prevotellaceae_UCG-003*), and for the strong correlation from *r* = 0.51 (*UCG-010*) to *r* = 0.66 (*Sphaerochaeta*). The exact results are shown in Fig. 3a.

### Generalised regression model

An analysis was conducted of how the experimental arrangement affected the microbiota at the phylum, genus and species level. The results are shown in Table 1. It was observed that the dose of microplastic was responsible for a change in the size of one population at the phylum level (Desulfobacterota) in 34.28%. In 14 genera, between 17.12 and 38.44% of the variability was caused by exposure to microplastic, and in the case of two bacteria species, MP-PET was responsible for the variability in 17.36–32.95%. The greatest impact was noted for *Coprococcus* (38.44%), *Sphaerochaeta* (37.73%), *Desulfovibrio* (35.72%) and *Treponema succinifaciens* (32.95%), and the smallest was for *Family XIII AD3011 group* (17.12%), *UCG-004* (17.18%), *Prevotella copri* (17.36%) and *Family XIII UCG-003* (17.83%).

**Table 1:** Generalised regression model showing statistically significant variabilities in microorganism populations depending on the MP-PET dose.

Dependent variable	Adjusted *R*^*2*^	*MS*	*SS*	*df*	*MS*	*F*	*p*
Phylum							
Desulfobacterota	0.34	768,785	3,308,242.83	26	127,240	6.04	0.00
Genus							
* Collinsella*	0.24	584,285	3,741,545.00	26	143,906	4.06	0.02
* Senegalimassilia*	0.23	243	1621.30	26	62	3.90	0.02
* Bacteroides*	0.23	447,927	2,956,683.64	26	113,719	3.94	0.02
* Rikenellaceae_RC9_gut_group*	0.25	3,691,334	22,324,781.93	26	858,645	4.30	0.01
* Desulfovibrio*	0.36	500,771	2,043,575.07	26	78,599	6.37	0.00
* Streptococcus*	0.21	281,973,972	2,068,954,888.99	26	79,575,188	3.54	0.03
* UCG-004*	0.17	1262	10,921.04	26	420	3.01	0.05
* Family_XIII_AD3011_group*	0.17	789,022	6,846,340.73	26	263,321	3.00	0.05
* Family_XIII_UCG-003*	0.18	11,895	99,866.01	26	3841	3.10	0.04
* Coprococcus*	0.38	1,434,033	5,299,613.24	26	203,831	7.04	0.00
* UCG-009*	0.30	294,995	1,498,855.33	26	57,648	5.12	0.01
* V9D2013_group*	0.21	2288	16,509.85	26	635	3.60	0.03
* UCG-010*	0.29	1,628,336	8,585,584.02	26	330,215	4.93	0.01
* Sphaerochaeta*	0.38	6394	24,238.52	26	932	6.86	0.00
Species							
* Prevotella_copri*	0.17	27,805	238,553	26	9175	3.03	0.05
* Treponema_succinifaciens*	0.33	588,601	2,661,259	26	102,356	5.75	0.00

The taxa which achieved *p* < 0.05 were taken into account at the phylum, genus and species level. Adjusted *R*^*2*^—coefficient of determination, *MS*—mean squares, *SS*—deviation of observed values from expected values, *df*—degrees of freedom, *F*—significance F, *p*—*p* value.

## Discussion

The impact of microplastics on higher organisms is a complex issue that requires continuous research. However, there is evidence that microplastics can have an adverse impact on the health and development of animals and humans^41,45,46,49,55,56^. One of the major challenges in designing research models is selecting the appropriate dose. Frequently, the doses taken seem to be overestimated in relation to the real exposure. The dose of 0.1 g/day used was selected based on the assumptions presented by Senathirajah et al. about the estimated amount of microplastic particles ingested by humans (0.1 g and 5 g of microplastics/week/person)^57^. The second experimental group (HD) were given the same MP-PET mixture at a dose ten times greater—1 g/animal/day. According to the data, pigs were exposed to 0.7 g/week and 7 g/week during the week. This exposure dose seems to be realistic. Using a mixture of particles of different sizes also more accurately reflects the environmental hazard.

Exposure to microplastic brought about changes in the microbiota composition in mice and chickens^46,48,58^. Examinations of samples collected from humans show that microplastic can have an impact on the composition and diversity of microorganisms present in the human colon^50^ or one may not note a considerable impact on the general microorganism population but only observe the presence of genes that encode plastic decomposing enzymes^59^.

MP-PET does not remain neutral to the pig microbiome. The changes in alpha diversity noted in this experiment can result from the ability to adapt to the new environmental conditions, stress caused by a change of location, a person looking after an animal, drinking water or social relationships between animals in newly formed groups^38,60–64^. The microbiome modulations resulting from environmental or zootechnical changes are natural phenomena. It is intriguing that they were not observed in the HD group. Since the animals originated from the same farm, it may be assumed that the observed differences result from individual characteristics, and a longer acclimatization period would be required to standardize the microbiome. Studies have indicated that in young animals, the intestinal microflora stabilizes around the age of 6 months^65^, during the fattening period, which ranges from 128 to 179 days of life^66^, or between 28 and 70 days of life^67^. Therefore, it’s not possible to definitively determine the age at which the stabilization occurs. Correlations between diversity and animal growth rate have also been established^67^. The observed differences among individuals from different groups on day 0 may result from the use of young animals for the experiment. Additionally, one has to strictly take into consideration individual factors, that would have to be disregarded when a study is performed on a larger population of animals. Due to restrictions on the use of animals in scientific research, the group size was limited to n = 5. For this reason, if possible, subsequent studies should be conducted on a larger research group, which will minimize individual characteristics. The results of statistical analyses are characterized by high standard deviation values, which only confirm the thesis that the overall group result is influenced by individual differences. Since the environment can be one of the microbiome-modulating factors^62^, it was to be expected that the microbiota compositions in groups C0, LD0 and HD0 would be highly similar, due to common origin. It can be assumed that the pigs from the HD group were already characterized by a stabilized microbiome, in contrast to the pigs from the C group. Inferences based solely on the alpha diversity results may lead to erroneous conclusions. An analysis of the microbiome composition at the different levels show differences between measurements made at the beginning and at the end of the experiment, although only subsequent statistical analyses show the taxa for which the exposure to microplastics was responsible for the changes. The results of the ANOVA with repeated measures show a higher number of reads for Firmicutes and *Oscillospiraceae* in group HD and for *Prevotella copri* in group C compared to the other two groups at the beginning of the study. This may be attributed to the application of the NGS method, whose undoubted advantage is its ability to identify more unique species than in traditional culturing methods. This can also be a disadvantage—the isolation of an excessive number of taxa^68^. However, the most likely reason for the observed differences at day 0 between groups, is the age of the animals used.

Microplastic may have created an adverse environment in the gut, thereby limiting the development of species abundance. Studies on diversity indices in chickens have shown no statistically significant changes in diversity indices (Simpson or Shannon), whereas the abundance indices (ACE and Chao1) indicated a decreasing trend^48^ unlike the findings of this study. However, it should be noted that, contrary to the study on chickens^48^, no changes in body weight were observed in pigs (Fig. 1i). A high microplastic dose may have impaired the microbiome’s ability to adapt to the new situation. Another explanation may be that comparable values of the mentioned indicators were recorded in the C and LD groups at the beginning of the experiment, which were lower than the values in the HD group.

An analysis of the research conducted by other authors has confirmed that Firmicutes and Bacteroidota are two main phyla of pig microflora^69–71^. Among other microorganism, Firmicutes are responsible for maintaining energy balance^67^. Bacteroidetes are responsible for maintaining digestive tract homeostasis, among other functions, by producing butyrate, which modifies the immune response and prevents the proliferation of pathogenic bacteria^67^. The Firmicutes/Bacteroidetes (F/B) ratio may serve as a marker for dysbiosis, obesity, or inflammation^72–74^. Studies have shown that this ratio increases with age and in obese animals in pigs^65,74^, with similar changes observed in humans^75^. In the presented studies, the mentioned ratio, calculated based on relative abundance, is as follows: C0—3.01, C28—5.16, LD0—3.51, LD28—3.57, HD0—4.26, and HD28—3.78. It can be observed that in pigs from group C, the F/B ratio increased with weight gain and age. The values obtained on day 0 and 28 indicate dysbiosis, because it is considered, that the dysbiosis index of < 2 is a normal value^51^. Interestingly, based on the dysbiosis index value, it can be observed that it increased in the control group. A higher dose of MP-PET could have contributed to alleviating this condition. In mice or humans, the F/B ratio was observed to increase^51,76^, which suggests the induction of dysbiosis under the influence of MP.

Studies in mice have shown that PS microplastics also contribute to reducing the Firmicutes population^58,77^. During exposure to 50 µm MP, a greater difference in relative abundance was noted compared to 0.5 µm particles^58^. Interestingly, a lower concentration (100 µg/L) caused a greater statistical difference (*p* < 0.01), than a concentration 10 times higher (*p* < 0.05)^58^. The results of Lu et al. correspond with the results obtained in this study, where a lower dose also contributed to a reduction in relative abundance by 4.64% and a higher one by only 0.67%^58^. This confirms the thesis, that smaller particles may potentially be more harmful, than larger ones and cause more negative effects. In the cited study, it was also observed that 50 µm MP particles caused a reduction in the Actinobacteria population, but 0.5 µm did not^58^. The correlations in pigs between MP-PET dose and Actinobacteriota showed moderate negative correlation. The obtained data are important because Actinobacteria are considered microorganisms that demonstrate the ability to biodegrade MP^53^. Their increased or decreased population provides insight into whether the microbiome is able to effectively neutralize MP.

It was shown in the subsequent statistical analyses, that a higher MP-PET dose increased the population size of *the Family XIII AD3011 group, Coprococcus, V9D2013 group*, and *UCG-010* of Firmicutes. An analysis of specific species shows that the population of *Megasphaera elsdenii* decreased in each group. However, statistical analyses did not show MP-PET to be responsible for this. *Megasphaera*, including *Megasphaera elsdenii*, are lactic acid fermentation bacteria which produce short-chain fatty acids (SCFA), such as acetate, propionate, butyrate and valerate, which are energy sources for animals, reinforce the epithelial barrier and alleviate inflammations in the intestines^78,79^. Potentially, SCFA could mitigate the negative effects of MP in the gastrointestinal tract. The results of studies using various types of MPs (PE, PCV, PS) indicate differences in the decrease/increase of the relative abundance of different bacteria, which may suggest, that the material from which the microplastic is made could influence the obtained results^80^. However, generally, it can be observed that MPs of different origins contribute to a decrease in the relative abundance of Firmicutes^48,80^ or Bacteroidetes^59^. The study by Lu et al., on the other hand, demonstrated that depending on the measurement day, the composition of the microbiome varies within weekly intervals^58^, which only confirms the hypothesis of the microbiome’s high adaptability and the possibility of significant dynamics in occurring changes. Analyzing the trend of changes at weekly intervals, rather than just measurements at the beginning and end of the experiment, could provide additional information and give a better overview of microbiome fluctuations.

An analysis of the class Bacteroidota did not show any statistically significant difference in this research model, unlike in the case of *Prevotella*. The relative population size of Bacteroidota increased in the HD group and decreased in the other two groups. The *Bacteroides* count can decrease with age^71^ and with an increasing body weight^69^. The statistical analyses showed that MP-PET was responsible for 17.36% of the *Prevotella copri* population size changes. Namely, the population in groups receiving microplastic on day 0 and day 28 was relatively constant, unlike in group C, where it was found to decrease. *Prevotella* is not usually regarded as a pathogenic genus, but recent evidence suggests that it may play a role in causing intestinal inflammation and subsequent release of substances, which can exacerbate the patient’s condition by developing anxiety and depression^63^. *Prevotella* exhibits the ability to decompose mucin, and it is responsible for the immunity and growth indices in pigs^81^. It is also one of the main genera producing SCFA^71,82^.

The statistical analysis (*r*-Pearson correlation and GRM, where *p* < 0.05) reveals certain microbe populations which could be affected by the microplastic dose: *Family XIII AD3011 group, Coprococcus, V9D2013 group, UCG-010* and *Sphaerochaeta*. The populations of all these microorganisms increased with increasing MP-PET dose. Changes in the bacteria population size may be linked to the developed response to the presence of microplastics in the gastrointestinal tract. Moreover, changes in microorganism populations take place mainly in taxa responsible for SCFA production. This is important because SCFAs are considered metabolites that prevent pathogen colonization, reduce oxidative stress and alleviate inflammation^48^. The induction of oxidative stress is one of the main mechanisms of MP’s impact on organisms^41^. An increased presence of the *Family XIII AD3011 group* can be correlated with tryptophan metabolism, by bacteria in the caecum and the colon, and with the production of indole or skatole, which can be toxic to spleen and lung cells, and can be markers for liver diseases^83^. A higher relative population size of the *Family XIII AD3011 group* in Tibetan pigs than in Yorkshire pigs was linked to improved immunity and more intensive fat deposition^84^. This confirms reports that the composition of the microbiome is also influenced by breed. Bacteria of *Coprococcus* and the *V9D2013 group* can be regarded as beneficial, because they are responsible for the production of butyrate^79,85^. This compound is responsible for maintaining the gut barrier integrity, restricting proinflammatory cytokine production, and inhibiting oncogenic pathways^79,86^. A reduced relative population size of *Megasphaera elsdenii* in the intestines, caused by the environmental conditions, may have induced an increase in the population size of other butyrate-producing bacteria, e.g. *Coprococcus* and *V9D2013 group* in order to maintain the homeostasis in the intestines in response to exposure to microplastic. An increased population of *Sphaerochaeta* in chickens was linked to an increased expression of fat catabolism genes, which decrease its deposition in the abdominal cavity while contributing to muscle growth^87^. These bacteria were linked to weight loss in horses^88^. Such changes have not been observed in pigs.

No reports have been found concerning *Oscillospirales UCG-10*, but it is known that *Oscillospira* can produce SCFA and is regarded as a new-generation probiotic^89^. It has been observed in this study, that a high dose of MP-PET contributed to maintaining a relatively constant level of the family *Oscillospiraceae* compared to a low dose. However, further analysis revealed a strong positive correlation between *Oscillospirales UCG-10* and a microplastic dose. It can be concluded that *Oscillospirales UCG-10* is one of the bacteria that is involved in the response to microplastics. This may be important in the selection of appropriate bacteria for the production of probiotics, the function of which would be based both on alleviating inflammation but also on the degradation of MP.

This study did not show any significant differences in the microbiome composition caused by microplastic in group HD based on the alpha and beta diversity indices, but, individual taxa were observed whose change was correlated with the dose. Nugrahapraja et al. put forward the hypothesis that microplastics must reach a specific level to cause perceptible changes in the host’s microbiome composition^59^. Further studies are required to determine this level, and the results may vary depending on the research model. A similar hypothesis could be based on this study, but changes were observed in groups C and LD, which can be indicative of the adaptive abilities of the microbiome and the MP-PET properties which inhibit these abilities.

It should also be considered that these are the first studies employing a research model with a high body weight (> 20 kg). For comparison, the estimated total surface area of the small intestine in a pig is 168–210 m^2^, whereas in a rat, it is 1.6 m^2^^90^. This might favor the accumulation of MP in the initial sections of the gastrointestinal tract, preventing them from reaching the large intestine, thereby explaining the absence of differences noted, as observed in studies on rats or mice^44–47,58^. However, this remains a hypothesis yet to be confirmed, as the accumulation of microplastics in the digestive tract has not been the subject of research. The similarities observed in groups C and LD may suggest that the dose of 0.1 g/animal/day is insufficient for the surface area of the digestive tract, and the observed changes merely represent a response to the new environment. It is also plausible that the material used in the experiment may exhibit less pronounced changes compared to the results of other authors utilizing a different type of plastic.

Moreover, it is noteworthy, that the more sensitive the method, the more changes may be caused by exposure to microplastics^49,91^. The presence of MP-PET particles in extracellular serum vesicles and the ability to affect the miRNA, associated with insulin resistance, obesity and cancers^91^, was demonstrated. Detecting these changes may not have noticeable consequences due to the corrective mechanisms and generalised homeostasis. Due to the labile nature of the microbiome, the organism can be regarded as striving to reach a balance. This study suggests that microplastics may be disrupting this process.

## Conclusions

The obtained results may suggest, that the environment had a significant impact on the observed differences in the composition of the faecal microbiome of domestic pigs. Under the influence of 28 days of oral exposure to MP-PET at a dose of 0.1 g/day and 1 g/day, changes were observed in the following taxa: *Family XIII AD3011 group, Coprococcus, V9D2013 group, UCG-010* and *Sphaerochaeta*. The above-mentioned taxa are mainly responsible for the production of SCFA. Due to their neuroimmunoendocrine function, it may be assumed, that short-chain fatty acids are one of the mechanisms involved in the response to oral exposure to MP-PET.

The obtained results require confirmation using other research methods, such as cytotoxicity studies, gene expression or changes in physicochemical properties of microparticles under the influence of digestion. In order to eliminate the influence of individual characteristics, it would be advisable to increase the research group and compare different age groups.

## Methods

### Microplastics

The polyethylene terephthalate (cat. no. ES306031/1, Goodfellow Cambridge Ltd., England) was analyzed using dynamic light scattering to determine the particle size distribution and microscopic analysis was used to visualize the shape and appearance of the particles.

**Fig. 4:**
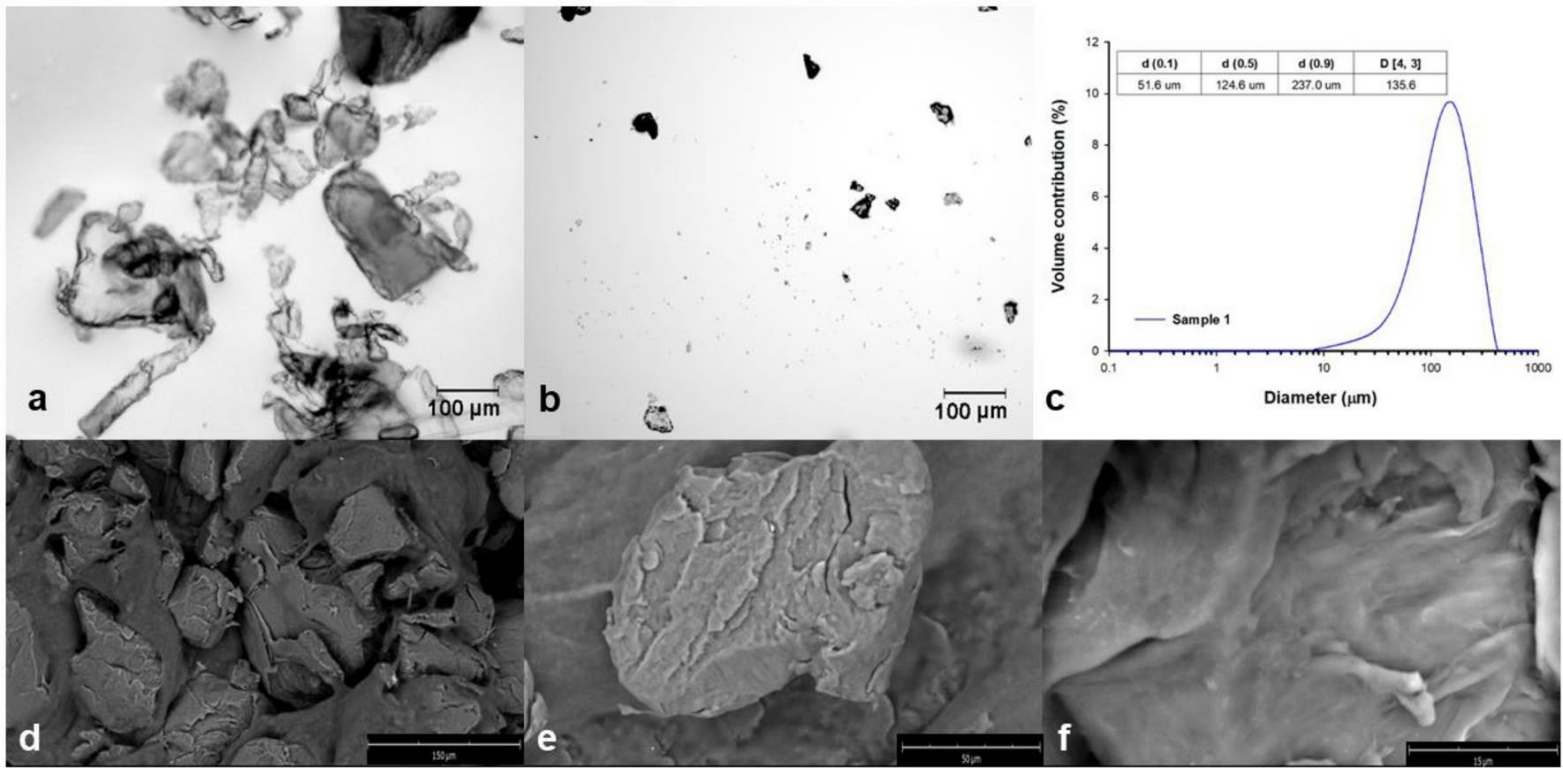
Characteristics of microplastic. (**a**, **b**) microscopic images, (**c**) particle size distribution and (**d**–**f**) SEM images of the polyethylene terephthalate.

Particle size was determined using a Mastersizer 2000 analyzer (Malvern Instruments Ltd., UK). The measurement was carried out using the dry method using the Scirocco 2000 device. A specific amount of sample (10–15 g) was introduced into the device. The measurement was performed with the following device settings: vibration intensity of the sample tray—25–30%, medium negative pressure in the measuring element 1.2–1.5 mBa. The obtained results indicate that the analyzed sample contains particles in the range of 7.6–416.9 µm, with the dominant share of particles with a diameter of 158.5 µm. Analysis of the results shows that in this sample 10% of particles have diameters not greater than 51.6 µm, 50% have diameters not greater than 124.6 µm, and 90% have diameters smaller than 237.0 µm. The mean diameter of particles D (4, 3) in the sample is 135.6 µm (Fig. 4c).

Microscopic analysis of particles was performed using a Zeiss Axio Imager.M2 (Zeiss, Oberkochen, Germany; Fig. 4a,b) and a Phenom ProX G6 scanning microscope (ThermoFisher Scientific, Waltham, MA, USA; Fig. 4d–f). Analysis of the results showed that the particles had different shapes and did not have a smooth surface.

### Experimental animals and procedures

The experiment was conducted on 15 sexually immature gilts aged eight weeks, weighing about 20 kg (Fig. 1i) of the Pietrain x Duroc breed from the farm in Lubawa (Poland). The animals were weaned at the age of 28 days. The animals were kept in the standard laboratory conditions (20–22 °C, 55–60% RH, 12 h/12 h light-darkness cycle), fed twice daily with commercial fodder, and had access to water ad libitum. Before the experiment, the rooms and the equipment were washed to remove potential biofilm and were disinfected with Virkon S (Bayer, Leverkusen, Germany), as recommended by the manufacturer, at a concentration of 1:50, exposure time of 30 min. The animals were randomly divided into three experimental groups (n = 5/group). The control group (C) were given empty gelatine capsules (Proton Labs Sp. z o.o., Gdańsk, Poland) once daily for 28 days. The first experimental group (LD) were given gelatine capsules with MP-PET particles at dose 0.1 g/animal/day.

The capsules were given one hour before the morning feeding. All the plastic objects were removed from the animals’ surroundings. The animals went through a 7-day acclimatisation period before the experiment. The body weight was measured every week, starting with the first day of the animals’ stay. The last body weight measurement was performed on the last day of the experiment.

All procedures were based on the approval given by the Local Ethical Commission (decision No. 10/2020 of 26 February 2020) and in accordance with the Polish law, which sets out the conditions and methods of conducting animal experiments in connection with the Act for the Protection of Animals for Scientific or Educational Purposes of 15 January 2015 (Journal of Laws 2015, no. 266), applicable in the Republic of Poland and Direvtive 2010/63/EU of the European Parliament and of the Council of 22 September 2010 on the protection of animals used for scientific purposes. The study was carried out in compliance with the ARRIVE guidelines.

### Microbiome examination

In order to determine the microbiota composition, individual samples of fresh faeces from gilts were collected on day 0 (before the capsule administration was started) and after 28 days of exposure to MP-PET. Immediately after the samples were gathered, they were frozen and kept at − 80 °C until they were used. Subsequently, the genomic DNA was isolated, as per the instructions, with a commercial kit (Genomic Mini AX Stool, A&A Biotechnology, Gdańsk, Poland). The concentration and purity of DNA (expressed by the ratio of absorbance at 260 nm and 280 nm (A260/A280)) were determined with a NanoDrop spectrophotometer (ThermoFisher Scientific, Waltham, MA, USA). The obtained material was kept at a temperature of − 20 °C until the metagenomic analysis was conducted with the next generation sequencing (NGS). The metagenomic analysis of the bacteria and archaea was based on the hypervariable V3–V4 region in the 16S rRNA gene. Amplification of the selected region and the preparation of the library was performed with specific primer sequences 341F and 785R (analysis 16S). The chain polymerase reactions were performed with Q5 Hot Start High-Fidelity 2X Master Mix (reaction conditions as per the manufacturer’s recommendations, New England Biolabs, Ipswich, MA, USA). The sequencing was performed on a MiSeq apparatus using paired-end (PE) technology, 2 × 300 nt, with a v3 Illumina kit. The automatic preliminary data analysis was performed on a MiSeq sequencer (Illumina, San Diego, Kalifornia, USA) with MiSeq Reporter (MSR) v2.6 software.

### Bioinformatic analysis

The bioinformatic analysis that ensured the classification of readings to the species level was performed with the QIIME 2 software package based on the reference sequence database Silva 138. The Divisive Amplicon Denoising Algorithm 2 (DADA2) package was also applied, which allowed for isolating sequences of biological origin from those newly created in the sequencing process. This package was also used for isolating the amplicon sequence variant (ASV). Quality control of the reads involved analyzing the error profile of individual samples and dynamically generating quality control parameters based on the maximum expected errors. Adapter sequences and reads shorter than 30 nucleotides were removed. Denoising, paired-end read pairing, dereplication, and chimera filtering were executed. Amplicon sequence variants (ASVs) or Operational Taxonomic Units (OTUs) were clustered with a similarity cutoff of ≥ 97%. Taxonomy was assigned to the ASV/OTU sequences utilizing the Silva 138 reference database. All the sequenced data were analysed in the R language. The QIIME 2 software was used to determine the alpha and beta diversity indices. The alpha diversity in all the samples was determined with the ACE, Chao1 and the observed species index representing abundance, and the Shannon and Simpson index representing diversity. The impact of MP-PET was determined by means of a beta diversity analysis based on the Bray–Curtis measures Principal Component Analysis (PCA) and Principal Coordinate Analysis (PCoA) were performed. The data were visualised with the ggplot2 package.

### Statistical analysis

The results were divided into six groups (C0—control group day 0, C28—control group day 28, LD0—low dose group day 0, LD28—low dose group day 28, HD0—high dose group day 0, HD28—high dose group day 28). To evaluate the distribution of alpha diversity, a one-way nonparametric Wilcoxon test (Simpson index) and Welch Two Sample t-test (ACE index, Chao1 index, Observed index and Shannon index) were used. The diversity indices were shown as *Mean* ± *SD*. The variability measurement between samples was performed with the beta diversity based on the Bray–Curtis distance. The beta diversity was assessed with a permutational multivariate analysis of variance (PERMANOVA) with 999 permutations with a vegan package.

Relative abundance was calculated based on the percentage of the number of counts of a given ASV sequence in relation to the sum of counts of all ASVs in a given group and expressed as a percentage. Sequences that were not assigned to bacteria were removed. This served to reduce noise for downstream analysis. Only those taxon readings which were noted in all the groups in at least one individual were taken for further statistical analysis. The assumption of linearity and normality was verified before the statistical analysis. In order to verify the linearity, two-dimensional point graphs of the variables under analysis were generated. The assumption of normality was validated with histograms and normality graphs for the rest. The following were calculated for the results: mean (*M*), standard deviation (*SD*), and standard error of the mean (*SEM*). The homogeneity of variance was verified with Levene’s test. In order to demonstrate the statistically significant differences between the groups w oparciu o numer of readings of ASVs, an ANOVA for repeated measurements was performed, taking into account the effect of the dose and replicate.

In order to demonstrate the linear correlations between the MP-PET dose and the number of readings of ASVs, the *r*-Pearson Correlation Coefficient was used. Subsequently, the results were assessed with the generalised regression model (GRM). The aim of this model was to assess the impact of the research group (qualitative variable) and the MP-PET dose (quantitative predictor) on the variability of the number of microorganism population readings (dependent variables), taking into account the relationships between the selected microorganism populations.

The statistical analysis was performed with Statistica 13.3 software (TIBCO Software Inc., Palo Alto, USA). The results were regarded as significant at *p* < 0.05. They were presented graphically with GraphPad Prism 9.0.0 software. (GraphPad Software, Boston, USA).

## Supplementary Information

Supplementary Information 1.

Supplementary Information 2.

Supplementary Information 3.

## Data Availability

The datasets used and/or analysed during the current study are available in the repository, https://www.ncbi.nlm.nih.gov/bioproject/1170529 Accession PRJNA1170529.
