# Supplementary material for: Influence of selected dosages of plastic microparticles on the porcine fecal microbiome
Source: Sci Rep. 2025 Jan 8;15:1269. doi: 10.1038/s41598-024-80337-x (PMC11711237; doi:10.1038/s41598-024-80337-x)

Supplementary Figure 1. Beta diversity in all experimental groups.

(**a-d**) Principal Component Analysis and (**e-h**) Principal Coordinate Analysis for faecal microbiome based on the Bray-Curtis measures at (**a**, **e**) OTU (**b**, **f**) phylum, (**c**, **g**) genus and (**d**, **h**) species levels. A dot represents each sample, and different colours represent different groups. C0 – control group day 0, C28 - control group day 28, LD0 – low dose group day 0, LD28 – low dose group day 28, HD0 – high dose group day 0, HD28 – high dose group day 28.

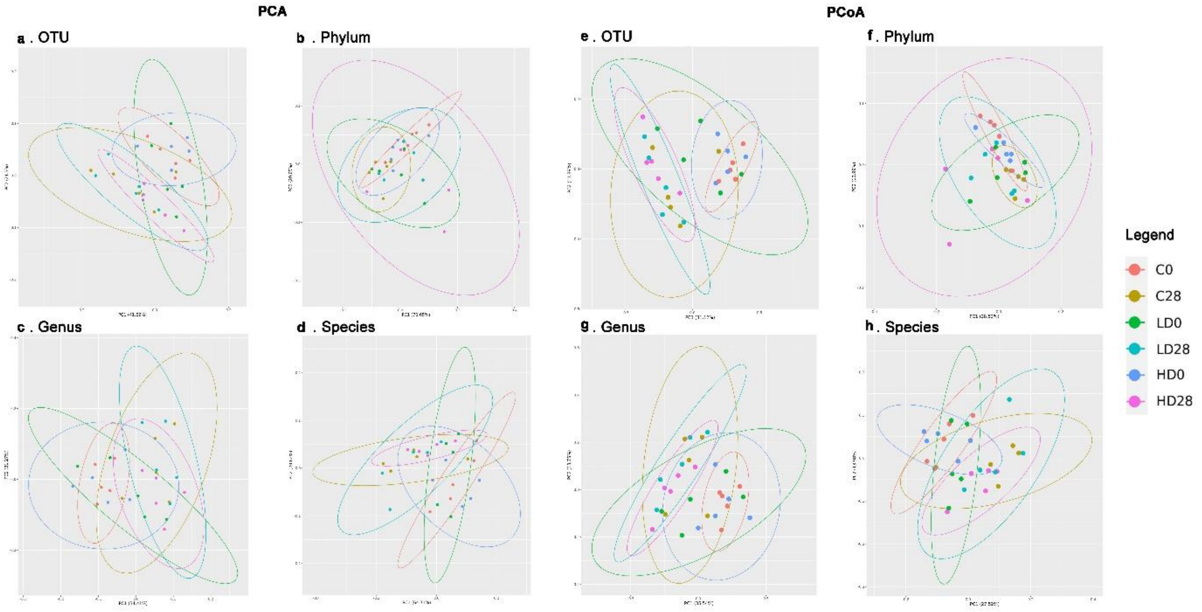

Supplement: Supplementary file 1 — Supplementary Information 1. [file 41598_2024_80337_MOESM1_ESM.pdf]
