# Supplementary material for: Influence of selected dosages of plastic microparticles on the porcine fecal microbiome
Source: Sci Rep. 2025 Jan 8;15:1269. doi: 10.1038/s41598-024-80337-x (PMC11711237; doi:10.1038/s41598-024-80337-x)
Supplement: Supplementary file 2 — Supplementary Information 2. [file 41598_2024_80337_MOESM2_ESM.docx]

|  |  | **Df** | **SumsOfSqs** | **F.Model** | **R^2^** | ***p*-values** |
| --- | --- | --- | --- | --- | --- | --- |
| **OTU** | | | | | | |
| C0  vs. HD28 | Group | 1 | 0.81284 | 5.5369 | 0.40902 | 0.008 |
|  | Residuals | 8 | 1.17444 |  | 0.59098 | ** |
|  | Total | 9 | 1.98729 |  | 1.00000 |  |
| C0  vs.  LD28 | Group | 1 | 0.51412 | 2.5587 | 0.24233 | 0.047 |
|  | Residuals | 8 | 1.60749 |  | 0.75764 | * |
|  | Total | 9 | 2.12161 |  | 1.00000 |  |
| C28  vs.  LD0 | Group | 1 | 0.58339 | 3.3321 | 0.29404 | 0.006 |
|  | Residuals | 8 | 1.40066 |  | 0.70596 | ** |
|  | Total | 9 | 1.98405 |  | 1.00000 |  |
| HD0  vs.  LD28 | Group | 1 | 0.41673 | 2.0695 | 0.20552 | 0.047 |
|  | Residuals | 8 | 1.61096 |  | 0.79448 | * |
|  | Total | 9 | 2.02769 |  | 1.00000 |  |
| HD28 vs.  LD0 | Group | 1 | 0.73331 | 4.3838 | 0.35399 | 0.011 |
|  | Residuals | 8 | 1.33824 |  | 0.64601 | * |
|  | Total | 9 | 2.07155 |  | 1.0000 |  |
| **Phylum** | | | | | | |
| C28  vs.  LD0 | Group | 1 | 0.10861 | 3.273 | 0.29034 | 0.029 |
|  | Residuals | 8 | 0.026547 |  | 0.70966 | * |
|  | Total | 9 | 0.037408 |  | 1.0000 |  |
| **Genus** | | | | | | |
| C0  vs. HD28 | Group | 1 | 0.39284 | 6.4297 | 0.44559 | 0.005 |
|  | Residuals | 8 | 0.48878 |  | 0.55441 | ** |
|  | Total | 9 | 0.88163 |  | 1.0000 |  |
| C0  vs.  LD28 | Group | 1 | 0.30137 | 4.0013 | 0.33341 | 0.033 |
|  | Residuals | 8 | 0.60253 |  | 0.66659 | * |
|  | Total | 9 | 0.90390 |  | 1.0000 |  |
| C28  vs.  LD0 | Group | 1 | 0.27133 | 3.6839 | 0.3153 | 0.01 |
|  | Residuals | 8 | 0.58923 |  | 0.6847 | * |
|  | Total | 9 | 0.86056 |  | 1.0000 |  |
| HD28  vs.  LD0 | Group | 1 | 0.33932 | 4.4245 | 0.35611 | 0.007 |
|  | Residuals | 8 | 0.61353 |  | 0.64389 | ** |
|  | Total | 9 | 0.95285 |  | 1.0000 |  |
| **Species** | | | | | | |
| C0  vs. HD28 | Group | 1 | 0.067678 | 5.0264 | 0.38586 | 0.018 |
|  | Residuals | 8 | 0.107717 |  | 0.61414 | * |
|  | Total | 9 | 0.175395 |  | 1.0000 |  |
| C28  vs.  LD0 | Group | 1 | 0.101735 | 11.2 | 0.58333 | 0.013 |
|  | Residuals | 8 | 0.072668 |  | 0.41667 | * |
|  | Total | 9 | 0.174403 |  | 1.0000 |  |
| HD28 vs.  LD0 | Group | 1 | 0.071456 | 6.2667 | 0.43926 | 0.015 |
|  | Residuals | 8 | 0.091220 |  | 0.56074 | * |
|  | Total | 9 | 0.162676 |  | 1.0000 |  |

**Table S1. Beta diversity analysis performed using PERMANOVA at the OTU, phylum, genus and species levels. Differences between groups, where **p*<0.05, ***p*<0.01. C0 ‑ control group day 0, C28 - control group day 28, LD0 – low dose group day 0, LD28 – low dose group day 28, HD0 – high dose group day 0, HD28 – high dose group day 28. Df – degrees of freedom, SumsOfSqs – sum of squares, F.Model – F value by permutation, R^2^ – explained variation, *p*-values based on 999 permutations.**
